# Supplementary material for: Mapping the evolution of fertility support policies in China: A content and instrumental analysis
Source: PLoS One. 2025 Oct 9;20(10):e0332137. doi: 10.1371/journal.pone.0332137 (PMC12510515; doi:10.1371/journal.pone.0332137)
Supplement: S1 Appendix — (ZIP) [file pone.0332137.s001.zip › S1 Appendix. 226 original policy documents/76-国务院关于女工作人员生产假期的通知(FBM-CLI-2-83).doc]

国务院关于女工作人员生产假期的通知

**发布部门：** [国务院](https://www.pkulaw.com/law/chl?Aggs.IssueDepartment=5)

**发布日期：**1955.04.26

**实施日期：**1955.04.26

**时效性：** [失效](https://www.pkulaw.com/law/chl?Aggs.TimelinessDic=02)

**效力级别：** [国务院规范性文件](https://www.pkulaw.com/law/chl?Aggs.EffectivenessDic=XC0203)

**法规类别：** [干部与工作人员](https://www.pkulaw.com/law/chl?Aggs.Category=00308) [劳动安全与劳动保护](https://www.pkulaw.com/law/chl?Aggs.Category=08706)

**失效依据：**本篇法规已被《女职工劳动保护规定》（发布日期：1988年7月21日 实施日期：1988年9月1日）废止

国务院关于女工作人员生产假期的通知
（一九五五年四月二十六日）

　　关于国家机关女工作人员生产假期，各地执行尚不一致。为避免相互影响，特统一规定如下：
　一、　生产：产前产后共给假五十六天，难产或双生增加假期十四天。

　二、　流产：怀孕不满七个月流产时，得根据医师的意见，给予三十天以内的产假。

　三、　产假期间（包括星期日及法定假日在内）工资照发。

　四、　产假期满，因病需要继续休养者，按病假处理。

© 北大法宝：（[www.pkulaw.com](https://www.pkulaw.com/)) 专业提供法律信息、法学知识和法律软件领域各类解决方案。北大法宝为您提供丰富的参考资料，正式引用法规条文时请与标准文本核对。
欢迎查看所有[产品和服务](https://www.pkulaw.com/helps/2.html)。法宝快讯：[如何快速找到您需要的检索结果？](https://www.pkulaw.com/helps/70.html)[法宝](https://www.pkulaw.com/helps/69.html)V6有何新特色？

扫描二维码阅读原文

**原文链接：**<https://www.pkulaw.com/chl/1209817908083dddbdfb.html>
